# Cost-effectiveness of vaccination with a quadrivalent HPV vaccine in Germany using a dynamic transmission model

**DOI:** 10.1186/2191-1991-2-19

**Published:** 2012-09-25

**Authors:** Deniz Schobert, Vanessa Remy, Oliver Schoeffski

**Affiliations:** 1 Lehrstuhl für Gesundheitsmanagement, Friedrich-Alexander-University Erlangen-Nürnberg, Lange Gasse 20, 90403, Nürnberg, Germany; 2 Market Access, Vaccine Advocacy & Medical Affairs, Sanofi Pasteur MSD, 8 rue Jonas Salk, 69007, Lyon, France

**Keywords:** Cervical cancer, Cervical intraepithelial neoplasia, Genital warts, Human papillomavirus, Quadrivalent HPV vaccine, Cost-effectiveness analysis, Dynamic transmission model

## Abstract

**Introduction:**

Persistent infections with human papillomavirus (HPV) are a necessary cause of cervical cancer and are responsible for important morbidity in men and women. Since 2007, HPV vaccination has been recommended and funded for all girls aged 12 to 17 in Germany. A previously published cost-effectiveness analysis, using a static model, showed that a quadrivalent HPV vaccination programme for 12-year-old girls in Germany would be cost effective. Here we present the results from a dynamic transmission model that can be used to evaluate the impact and cost-effectiveness of different vaccination schemas.

**Methods:**

We adapted a HPV dynamic transmission model, which has been used in other countries, to the German context. The model was used to compare a cervical cancer screening only strategy with a strategy of combining vaccination of females aged 12–17 years old and cervical cancer screening, based on the current recommendations in Germany. In addition, the impact of increasing vaccination coverage in this cohort of females aged 12–17 years old was evaluated in sensitivity analysis.

**Results:**

The results from this analysis show that the current quadrivalent HPV vaccination programme of females ages 12 to 17 in Germany is cost-effective with an ICER of 5,525€/QALY (quality adjusted life year). The incremental cost-effectiveness ratio (ICER) increased to 10,293€/QALY when the vaccine effects on HPV6/11 diseases were excluded. At steady state, the model predicted that vaccinating girls aged 12 to 17 could reduce the number of HPV 6/11/16/18-related cervical cancers by 65% and genital warts among women and men by 70% and 48%, respectively. The impact on HPV-related disease incidence and costs avoided would occur relatively soon after initiating the vaccine programme, with much of the early impact being due to the prevention of HPV6/11-related genital warts.

**Conclusions:**

These results show that the current quadrivalent HPV vaccination and cervical cancer screening programmes in Germany will substantially reduce the incidence of cervical cancer, cervical intraepithelial neoplasia (CIN) and genital warts. The evaluated vaccination strategies were all found to be cost-effective. Future analyses should include more HPV-related diseases.

## Background

Persistent infections with human papillomavirus (HPV) are a necessary cause of cervical cancer and are responsible for important morbidity in both men and women [1,2]. HPV are classified as high or low risk, based on their oncogenic potential. In Europe, high risk types HPV16 and HPV18 are responsible for 75% of cervical cancers, 60% of high-grade cervical intraepithelial neoplasia (CIN2/3) and 25% of low-grade cervical intraepithelial neoplasia (CIN1) [3,4]. Low risk types HPV6 and HPV11 are responsible for about 10% CIN1 and 90% of genital warts [5]. HPV are also responsible for some other anogenital cancers (vulvar, vaginal, anal and penile) and some head and neck cancers [6-8].

In Germany, it is estimated that there are nearly 5,500 new cases of cervical cancer and 1,500 cervical cancer deaths every year [9,10]. The EU-standardized incidence and mortality rates were estimated to be 11/100,000 and 2.5/100,000 women in 2006 [9]. Genital warts are frequent; the estimated incidence is 169.5/100,000 person-years for the German population aged 10 to 79 [11]. The peak incidence occurs in females at 20 to 24 years (627/100,000 person-years) and in males at 25 to 29 years (457/100,000 person-years) [11].

Cervical cancer is one of the target cancers covered by the statutory German cancer screening programme. All German women are eligible to receive an annual cervical examination including a Papanicolaou (Pap) smear, beginning at 20 years old. The annual uptake has been estimated to be about 50% of the eligible population [12].

Since 2007, HPV vaccination has been recommended and funded for all girls aged 12 to 17 in Germany [13]. Two prophylactic vaccines are currently available. One is a quadrivalent HPV6/11/16/18 vaccine, licenced for the prevention of premalignant genital lesions (cervical, vulvar and vaginal), cervical cancer and external genital warts [14]. The other vaccine is a bivalent HPV16/18 vaccine, licenced for the prevention of premalignant cervical lesions and cervical cancer [15]. In addition, the results from a randomised controlled trial in men demonstrated the efficacy of the quadrivalent HPV vaccine against external genital lesions in men, including genital warts and anal precancerous lesions [16,17].

A previously published cost-effectiveness analysis, using a static model, showed that a quadrivalent HPV vaccination programme for 12-year-old girls in Germany would be cost effective, with an incremental cost-effectiveness ratio (ICER) of 10,530€/QALY (quality-adjusted life year) [18]. However, this static model cannot take into consideration changes in HPV infection rate over time and it did not adequately reflect the current recommendation in Germany to vaccinate 12 to 17-year-old-girls. Dynamic transmission models, unlike static models, can be used to evaluate the epidemiological impact and cost-effectiveness of different vaccination schemas, taking into account both direct (for those vaccinated) and indirect effects (in those not vaccinated: herd immunity effect). This modelling approach will be useful for healthcare decision makers in estimating the expected benefits from the vaccination programme that has been implemented in Germany. In this paper we report the results from the adaptation of a previously published [19] dynamic transmission model to assess the health and economics impact of the quadrivalent HPV vaccine in Germany from a third-party payer perspective.

## Methods

### Dynamic transmission model

**Figure 1:**
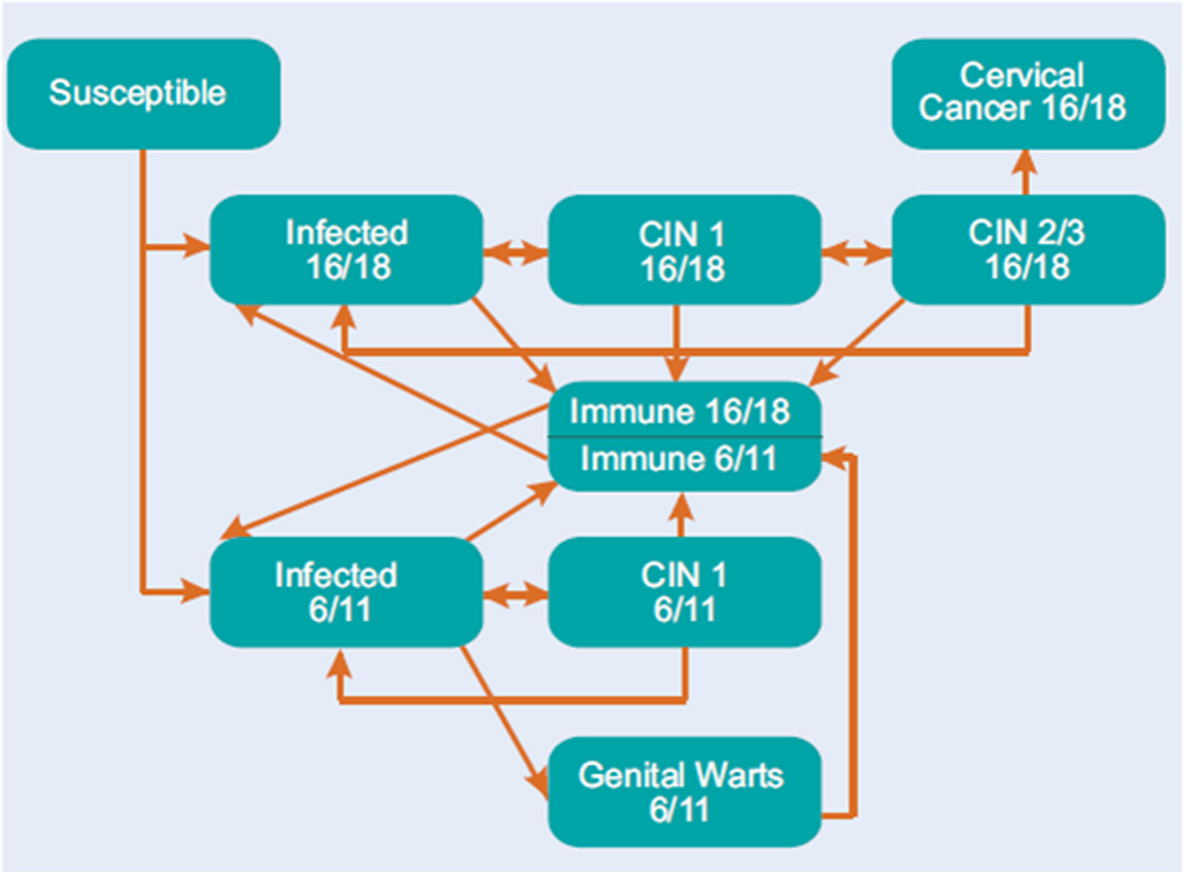
Schematic representation of the dynamic transmission model CIN: cervical intraepithelial neoplasia.

We adapted a HPV dynamic transmission model that has already been used in the United States (US), United Kingdom (UK), Mexico and Norway to the German context [19-22]. The details of the model structure and the equations have been published previously and are outlined in Figure 1[21,23]. In summary, this model simulates the spread of HPV 6/11/16/18 infection and diseases (CIN, cervical cancer, genital warts) in an age-structured population. The demographic portion of the model defines the characteristics of the simulated population and describes how people enter, age and exit the model. The epidemiological portion of the model simulates HPV transmission and infection as well as the development of the HPV related diseases in the sexually-active population of females and males.

The model outcome parameters were driven by five categories of input parameters: demographic data; sexual behaviour; cervical cancer screening; natural history; and treatment patterns.

### Adaptation process

There were three steps in the adaptation process:

1. Assessment of the transferability of the model structure to the German setting

2. Review of the input parameters to fit the German setting

3. Manual calibration of the model to fit the epidemiology data observed in Germany

### Transferability of the model structure

There is no evidence that the course of disease, from HPV infection passing through the various disease stages up to cervical cancer death, is different in Germany from that in the US or any other country. The original model also considers treatment probabilities instead of explicit treatment strategies. This makes the model easily transferable to other countries. Furthermore, the screening module in the original model is based on yearly routine cervical cancer screening for women over 20 years old which is consistent with the recommendation in Germany [24]. Thus, we concluded that the model structure is transferable to the German situation.

### Model input parameters

A literature search was undertaken to identify German-specific values for the input parameters; this search was supplemented by advice from experts and cancer registry analyses, when necessary.

### Demographic data and sexual behaviour patterns

The demographic data was obtained from the German federal statistical office; the total population aged 12 and over in Germany on 31 December 2008 was estimated to be 73 234 448 people [25]. No German-specific data were identified for the sexual behaviour patterns. Since the data in the literature suggested that the sexual behaviour patterns are similar to those in the UK, so these data from the UK were integrated into the model (Additional file 1: Table S1) [26].

### Cervical cancer screening and vaccination strategies

The annual cervical cancer screening rates and the sensitivity and specificity of Pap smear tests and colposcopy were based on data from a German health technology assessment report (Table 1) [10].

**Table 1:** **Cervical cancer screening parameters (based on**[10,27]**)**

**Parameter**	**Parameter estimate (%)**
% of women not regularly screened	40
Annual screening rates by age group	
12-14	0
15-17	0
18-19	0
20-24	54.62
25-29	55.93
30-34	53.90
35-39	52.09
40-44	50.29
45-49	49.51
50-54	48.80
55-59	46.94
60-64	43.76
65-69	37.63
70-74	27.50
75-79	19.26
80-84	9.02
>85	9.02

Based on clinical trial data, the prophylactic efficacy of the quadrivalent HPV vaccine for preventing incident HPV 6/11/16/18 infections was assumed to be 90% [28]. We assumed the efficacy of the quadrivalent HPV vaccine for preventing HPV 6/11/16/18-related CIN, cervical cancer and genital warts was 95.2%, 100% and 98.9% respectively [28]. Additionally, the model included the fact that the quadrivalent HPV vaccine would not protect against HPV disease present at the time of vaccination and that it would not affect the rate of clearance and infectiousness of ‘breakthrough’ cases [19]. In line with other models, we assumed in the base case that the quadrivalent HPV vaccine provided life-long protection [21,29].

The model was used to compare a cervical cancer screening only strategy with a strategy of combining vaccination of females aged 12–17 years old and cervical cancer screening, based on the current recommendations in Germany. The vaccination coverage rates were set to reflect the current situation in Germany for the first years of vaccination. In the base case, annual rates by age were set to reach a total cumulative coverage rate of 45% and 55% for the 12–14 and 15–17 respectively (Table 2). In addition, the impact of increasing vaccination coverage in this cohort of females aged 12–17 years old was evaluated in sensitivity analyses (Table 2).

**Table 2:** Annual vaccination coverage rate (%) in each year and by age group

**Years since start of the vaccination programme**	**Annual vaccination coverage rates (base case scenario, %)**			**Predicted increased vaccine coverage rates after six years of vaccination (sensitivity analyses, %)**		
	**12 year old cohort**	**13-14 year old girls**	**15-17 year old girls**	**12 year old cohort**	**13-14 year old girls**	**15-17 year old girls**
1	20	27	38	*NA*	*NA*	*NA*
2	14	28	33	*NA*	*NA*	*NA*
3	8	9	16	*NA*	*NA*	*NA*
4	8	11	6	*NA*	*NA*	*NA*
5	11	13	6	*NA*	*NA*	*NA*
6	12	14	7	*NA*	*NA*	*NA*
7	13	15	8	15	20	12
8	14	16	9	20	25	18
9	15	17	10	30	25	20
10	16	19	11	40	30	25
11+	16	19	11	45	30	25

*NA:* Not Applicable.

### Disease and treatment patterns

Natural history parameters were based on international data and were the same as in the original model [23]. Cervical cancer mortality rates by stage (local, regional, distant) and age were not publicly available for the whole of Germany as there is no central cancer registry. Therefore we used data from the Bavarian Cancer Registry and modified it to account for the difference in the cancer death rate between Bavaria and all of Germany (Additional file 1: Table S2) [32]. The cervical cancer mortality rates in Bavaria (world standardised rate of 1.6 per 100,000 women) are lower than expected for the whole of Germany (world standardised rate of 1.8 per 100,000 women) [30]. We, therefore, adjusted the data from Bavaria by 12.5% to reflect the estimated national rate.

The percentages of women treated for CIN/carcinoma in situ (CIS) and genital warts were based on German cost studies (Additional file 1: Table S3) [31]. The percentage of women, by age group, who undergo hysterectomy annually, was based on data from a German database (Additional file 1: Table S4) [24].

### Economic parameters

The costs for the diagnosis and treatment of HPV-related diseases were based on German cost studies and a German health technology assessment report (Table 3) [18,31]. The cost for three doses of vaccine and administration was set at €451.20 (Table 3).

**Table 3:** **Estimated costs and utility values by health states (Sources:**[18,31,33]

**Parameter**	**Estimated cost (€)**	**Utility scores**
Conventional cytology screening visit and test	24.80	
Colposcopy	23.60	
Biopsy	106.00	
Treatment for one episode:		
CIN1	336.00	0.91
CIN2	336.00	0.87
CIN3	1,498.00	0.87
Local cervical cancer	7,523.00	0.76
Regional cervical cancer	15,649.00	0.67
Distant cervical cancer	17,152.00	0.48
Cancer survivor	-	0.76
Treatment for GWs:		
males	550.00	0.91
females	550.00	0.91
Vaccination (3 doses and administration)	451.20	-

The health utility values for the disease (Table 3) and German general population (Additional file 1: Table S5) health states were based on published data [33,34].

### Model validation

The predictive validity of the model was assessed by comparing predictions from the model with available epidemiological data on incidence and mortality of cervical cancer and genital warts in Germany [11,30]. The epidemiologic data for genital warts, cervical cancer and cervical cancer deaths in Germany was adjusted by the percentage estimated to be HPV6/11/16/18-related (Table 4) [3,5]. A target window of ±10% was set to define how well all the calibration targets fitted.

**Table 4:** Calibration targets for genital warts (in females and males), cervical cancer cases and deaths

	**Genital warts (males and females)**	**Cervical cancer**	**Cervical cancer deaths**
Annual number of cases	117,431	5,470	1,492
Percentage HPV-related cases*	90	76.2	76.2
Adjusted number of cases	105,688	3,829	1,044
Defined ± 10% window	95,119 - 116,257	3,751 - 4,585	1023 - 1,251

* HPV6/11 for genital warts [5]; HPV 16/18 for cervical cancer cases and deaths [3,9,11].

The model was calibrated to ensure that predicted values were consistent with the expected epidemiological data for Germany. We did not modify the natural history parameters values used in the original US model as they were the results from an extensive calibration process and they are not expected to change between countries [21]. Calibration involved only a few parameters that were based on either imprecise or assumed data that are responsible for reduced reliability: we had to slightly modify annual cervical cancer screening rates, the proportion of women not regularly screened and cervical cancer mortality rates. Values finally used (presented in Table 1 and Additional Tables) were still considered as realistic for the German setting. We adjusted the model to obtain the target corridor sequentially to minimize the interdependency of the modified parameters: First, we adjusted for the annual number of genital warts, then the annual number of cervical cancer cases, followed by the annual number of cervical cancer deaths. This order was chosen because genital warts are considered to be independent of the other two parameters and cervical cancer incidence and cervical cancer deaths are correlated.

### Model analyses

The analyses were done from a third-party payer perspective, over a lifetime horizon. The discount rate on all costs and benefits was set at 3%. The model was used to assess the impact of HPV 6/11/16/18 vaccination on the burden of HPV 6/11/16/18-related diseases (cervical cancer and genital warts) in both males and females. The potential cross-protection effect of the vaccine against HPV types not included in the vaccine was not evaluated as these types are included in the model.

The model also provided an estimation of the quality-adjusted life years (QALYs), total costs and the incremental cost-effectiveness ratio (ICER).

Univariate sensitivity analyses were performed to identify which of the following parameters influenced the results most strongly:

1 Vaccine duration of protection (20 years)

2 Disease management costs (by +/− 20%);

3 Disease utility scores (+/− 20% and cancer survivor utility set to 1)

4 Discount rates at 0% and 5%;

5 No prevention of HPV6/11 diseases;

6 Increased vaccine coverage for women aged 12–17 years (table 2)

## Results

### Results of the validation process

The calibrated model predicted, with current screening practices and in the absence of vaccination, an annual number of 104,852 cases of HPV6/11 related genital warts in both males and females (target: 95,119 to 116,257); 4,507 cases of HPV16/18 related cervical cancer (target: 3,751 to 4,585) and 1,101 cervical cancer deaths (target: 1,023 to 1,251) (Table 4). Therefore, the predictions from the model fitted observed epidemiological data in Germany.

### Epidemiological impact of HPV6/11/16/18 vaccination in Germany

**Figure 2:**
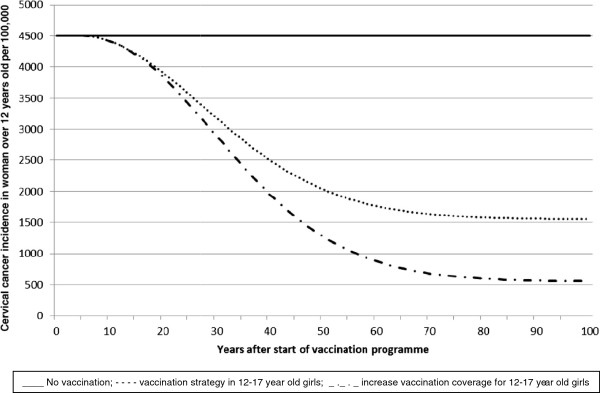
Effect of vaccination strategies on the incidence of cervical cancer.

The annual number of HPV6/11/16/18 events prevented with vaccination at different time frames are summarised in Table 5. Figure 2 shows the impact of vaccination over time on the incidence of HPV16/18 related cervical cancer cases. The decrease in the incidence of cervical cancer would be continuous and stabilise at approximately 1500 cases per year (65% reduction) at about 80 years after the start of the vaccination programme. After 25 years from the start of the programme, a 21% reduction in the incidence of cervical cancer would be seen. The impact of increasing vaccination coverage in this age group would lead to earlier reductions and result in a plateau after about 80 years at about 600 cases of cervical cancer cases annually, which is a reduction of 87%. The impact of the current vaccination programme on cervical cancer mortality also shows a continuous decrease to a plateau of about 380 deaths annually (reduction of 64%).

**Table 5:** Annual number of HPV 6/11/16/18 events prevented by quadrivalent HPV vaccination of 12 to 17 year old girls in Germany and screening, compared with screening alone

	**Annual number of disease events prevented**				
	**Time since start of vaccination programme (years)**				
	**5**	**15**	**25**	**50**	**100**
Cervical cancer	5	298	953	2,474	2,955
Cervical cancer deaths	0	21	135	559	720
CIN 2/3	1,711	9,288	12,694	14,899	15,429
CIN 1	899	3,552	4,366	4,751	4,829
Genital warts					
female	16,350	30,780	33,001	35,075	36,049
male	13,874	26,999	27,172	25,948	25,691
Total	30,225	57,779	60,173	61,023	61,740
HPV 16/18 events	2,322	12,134	16,876	20,906	21,966
HPV 6/11 events	30,518	58,783	61,309	62,241	62,987

A similar impact on the incidence of pre-cancerous CIN1 and CIN2/3 was predicted, with a 64% decrease with about 15,500 of CIN2/3 cases avoided annually and a 59% decrease with about 4,800 cases of CIN1 prevented annually in the long term. In a shorter term i.e. after 20 years from the start of vaccination, a 48% and 50% reduction was observed in CIN2/3 and CIN1, respectively. Increasing vaccination coverage would lead to earlier benefits (a 50% reduction in CIN1 being observed 4 years earlier i.e. after 16 years from the start of vaccination) and greater (87% and 81% reduction in CIN2/3 and CIN1 in the long term) reductions.

**Figure 3:**
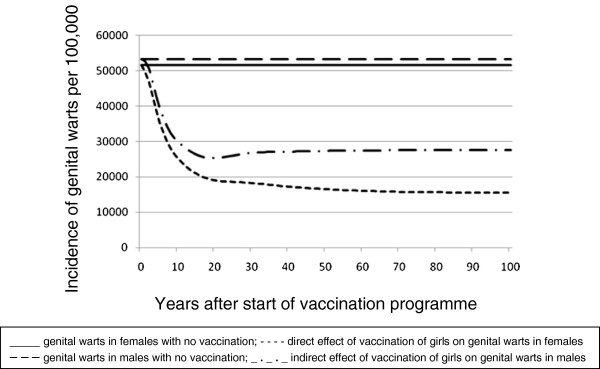
Effect of quadrivalent HPV vaccination in girls aged 12 to 17 years on the incidence of genital warts in females and males.

The impact on genital warts is seen within a short time because of their more rapid disease progression. After less than five years, it is estimated that the incidence of HPV6/11 related genital warts will be reduced by 25% (Figure 3). After 15 years, quadrivalent HPV vaccination would avoid 57,780 cases of genital warts in males and females (55% reduction). At steady state, genital wart cases among women and men (through herd effect) will be reduced by 69.9% and 48.2% respectively.

### Economic impact of HPV6/11/16/18 vaccination in Germany

Overall, the vaccination strategy would avoid 29% and 61% of the total discounted HPV6/11/16/18-related diseases costs at 15 years and long term, respectively. Due to the nature of the diseases, the discounted annual HPV 6/11/16/18-costs avoided show their peak for genital warts after 10 years, for CIN after 30 years and, finally, for cervical cancer after 40 years. In the first five years of vaccination, 96% of the total HPV-related diseases costs avoided would be attributable to the prevention of HPV6/11-related diseases, due to the generally shorter latency period for these diseases. This relative proportion would decrease to 44% after 50 years. Over 100 years, 57% of the total cumulated discounted costs avoided would be linked to prevention of genital warts.

### Cost-effectiveness and sensitivity analyses

The ICER for vaccination of 12–17 year-old girls in Germany was estimated at 5,525€/QALY and 10,205€/LYG (life-years gained) (Table 6), which is below the threshold commonly used in the UK (£30,000/QALY) by the National Institute for Health and Clinical Excellence (NICE) in economic evaluations as the threshold ratio. We used this threshold since in Germany, there is no fixed threshold of ICER below which health technology is considered as ‘good value for money’ [35].

**Table 6:** Cost-effectiveness of quadrivalent HPV vaccination in Germany

	**Costs (€)**	**Δ Costs (€)**	**QALYs**	**Δ QALYs**	**Δ Costs/Δ QALYs (€/QALY)**	**LYG**	**Δ LYGs**	**Δ Costs/Δ LYG (€/LYG)**
Screening only	15,108,454		2,853,042			3,163,556		
Base case	19,506,654	4,398,200	2,853,838	796	5,525	3,163,987	431	10,205

**Figure 4:**
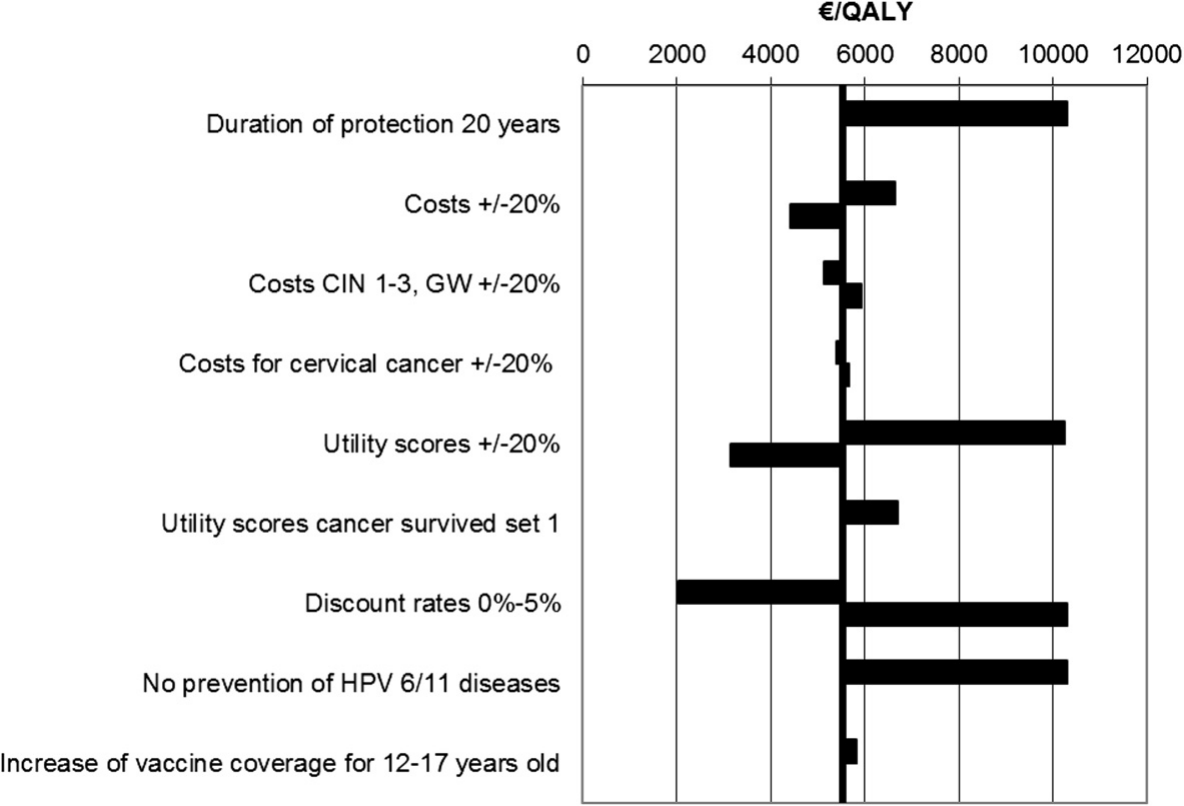
Tornado diagram summarising the results of the sensitivity analyses.

The results were most sensitive to discount rates, duration of vaccine protection and utility scores but remained below the NICE threshold of £30,000/QALY so that HPV vaccination in the German setting can be considered to be cost-effective (Figure 4). Excluding the protection against HPV 6/11 infections and diseases increased the ICER to 10,296€/QALY. Increasing vaccine coverage for 12 to 17 year old girls in Germany as described in sensitivity analyses only increased the ICER to 5,807€/QALY. Variation in disease treatment costs had a minor impact on the ICER (Figure 4).

## Discussion

The results from this analysis show that the current HPV vaccination programme of females ages 12 to 17 in Germany is cost-effective with an ICER of 5,525€/QALY. Excluding the vaccine effect on HPV6/11 diseases was considered, the ICER increased to 10,293€/QALY. While this remains cost-effective using the NICE threshold, it emphasises the added value of protection against HPV6/11 diseases. Increasing vaccine coverage in the recommended population would lead to larger and earlier reductions with only a small impact on the ICER (5,807€/QALY).

At steady state, the model predicted that vaccinating girls aged 12 to 17 could reduce the number of HPV 6/11/16/18-related cervical cancers by 65% and genital warts among women and men by 70% and 48% respectively. The impact on HPV-related disease incidence and costs avoided was found to occur relatively soon after initiating the vaccine programme, with much of the early impact being due to the prevention of HPV6/11-related genital warts. There is consistent and growing evidence from several studies that the effects predicted by this model are being observed in the real world. In Australia, in 2009, 65% of Australian females eligible for free vaccination (i.e. female Australian residents aged between 12 and 26 years old) had been vaccinated [36]. A study comparing two 12-month periods in 2007–08 and 2010–11 showed a dramatic reduction in the incidence of genital warts from 18.6% to 1.9% and 22.9% to 2.9% in under 21-year-old women and heterosexual men, respectively, attending a sexual health centre in Melbourne 4 years following the start of vaccination implementation [37]. The same trend was reported in another study of patients attending eight sexual health services in different states of Australia between January 2004 and December 2009 [36]. Since no other relevant health intervention was implemented during this time and the incidence of other sexually-transmitted diseases was unchanged the observed effect is most likely to be attributable to the broad and high vaccine coverage in young women. This is reinforced by similar results reported in New Zealand [38] and California.

Our model also predicts a reduction in cervical disease outcomes, which has been reported in two studies, so far. In a case–control study in New York, girls aged 11 to 21 who had received at least one HPV vaccination prior to the first Pap smear were reported to have a statistically significant lower risk of having an abnormal Pap smear result (OR = 0.254; 95%CI: 0.093-0.698; p = 0.008) [39]. Results from an ecological study suggest an early effect from the HPV vaccination programme on cervical abnormalities in Victoria, Australia [40]. They reported a decrease in the incidence of high-grade cervical abnormalities of 0.38% (95%CI: 0.61-0.16) in girls under 18 years old less than three years after the introduction of the quadrivalent HPV vaccination programme. This decline was not observed for low-grade cervical abnormalities or in older age groups [40].

Our results are consistent with those predicted using a static model for Germany which found an ICER of 10,530€/QALY for preventing cervical cancer and genital warts [18], although the two analyses did not compare the same vaccination cohort and the structures of the models are different.

The limitations of this model have been described in detail elsewhere but here we will briefly discuss some potential limitations specific to this analysis [21]. Firstly, as no German-specific data on sexual behaviour were available, we used data from the UK. The impact this may have on the predictions is unknown [26]. Secondly, we used a simple calibration approach, similarly to what was done in the Norwegian and UK adaptation, and we calibrated the model with global incidence but not with age-specific data [19,20]. It is clear that the calibration process is one of the critical steps of the model adaptation. The model was initially developed for the US and followed an extensive calibration approach, where age-specific data were considered. As we did not modify any natural history parameters, but only a few parameters such as the proportion of women who do not screen regularly, we probably did not changed the peak of the incidence curves of cervical cancer incidence and mortality, and we think that focusing on global incidence rates was an acceptable approach. Thirdly, we considered only direct costs; in future analyses, indirect costs, such as productivity loss, should be included so that the results would be closer to reality. Another limitation concerns the estimates for the health utilities, which were taken from a study performed in the US. However, as far as we are aware, there are no such estimates available for Germany or for any other European country. We also assumed 100% vaccine adherence with each of the three vaccine doses without making allowance for a drop-out rate after one or two doses which may not reflect the reality, but there is no efficacy data available for less than three doses. Furthermore, we did not consider any potential adverse effects of the vaccine. Although vaccination with the quadrivalent vaccine is generally well tolerated so we would expect that including them into the model would have only a minor impact on the ICER.

Additionally, we did not include potential vaccination impact on non-vaccine types in our model, as the model did not include HPV types non-included in the vaccines. There is uncertainty about the accuracy of the observed cross-protection as it is not always easy to determine the causal HPV type in a lesion [41]. It is also unknown whether cross-protective efficacy will be long lasting [41], recent data suggest that cross-protective effects are short lived and therefore of limited value. The results from a UK modelling analysis comparing the cost-effectiveness of the quadrivalent and bivalent vaccines, showed that additional cross-protection only had a minor contribution in terms of economic benefits (i.e. QALYs gained and costs prevented), especially if compared with benefits for genital warts [41]. Therefore, we could assume that inclusion of vaccine efficacy against non-vaccine types would only have a limited impact on our results.

Another limitation is that we did not perform probabilistic sensitivity analyses. Nevertheless, results from the sensitivity analysis confirm the findings of many other models and showed that results were sensitive to assumptions about the duration of protection and discount rates.

Lastly, only cervical diseases and genital warts were included in the present model. However, HPV has been found in significant numbers of vulvar, vaginal and anal cancers and the quadrivalent vaccine has demonstrated high efficacy in preventing these lesion [6,17,42]. HPV is also found in a proportion of head and neck squamous cell cancers [7]. Recently two reviews have concluded that the available data are consistent with the hypothesis that HPV does have a causal role in head and neck cancer [43,44]. There are no clinical efficacy data for head and neck cancer because there are no precursor lesions for head and neck cancer and recurrent respiratory papillomatosis (RRP) is very rare. However, it is reasonable to think that the quadrivalent HPV vaccine could prevent these diseases. If these diseases were to be included in the model, the protection by HPV vaccination would be expected to be greater, which would give a lower ICER. HPV-related non-cervical cancers were included in a recent UK modelling study that predicted that, in addition to a median of 700 to 1000 cervical cancers, in the long-term, HPV vaccination could prevent between 620 and 950 cases of these cancers annually in the UK [41]. Future cost-effectiveness analyses for HPV vaccination should, therefore, include these diseases to provide a more accurate prediction of the cost-effectiveness of HPV vaccination. In light of the recent clinical trial results showing the efficacy of the quadrivalent HPV for the prevention of genital warts and precancerous anal lesions in males it will also be of importance for decision makers to assess the potential impact of vaccination of females and males on female and male HPV-related diseases [16,17]. Some countries, e.g. Australia, Canada and the US have recently recommended the inclusion of boys in their vaccination programmes.

## Conclusions

The results from this model show that the current quadrivalent HPV vaccination programme, in addition to the screening programme in Germany, will provide public health benefits by substantially reducing the incidence of cervical cancer, CIN and genital warts. The vaccination strategies evaluated in this study were all found to be cost-effective. Future modelling analyses should include more HPV-related diseases.

## Abbreviations

CIN: Cervical intraepithelial neoplasia; CIS: Carcinoma in situ; HPV: Human papillomavirus; ICER: Incremental cost-effectiveness ratio; LYG: Life year gained; NICE: National Institute for Health and Clinical Excellence; Pap: Papanicolaou test; QALY: Quality-adjusted life year; RRP: Recurrent respiratory papillomatosis; UK: United Kingdom; US: United States.

## Competing interests

DS and OS are employees at Friedrich-Alexander-University Erlangen-Nürnberg, which received an unrestricted grant from Sanofi Pasteur MSD.

VR is employed by Sanofi Pasteur MSD. 

The authors have no other relevant affiliations or financial involvement with any organisation or entity with any financial interest in or financial conflict with the subject matter or materials discussed in the manuscript apart from those disclosed.

## Authors' contributions

All authors satisfied the ICMJE authorship criteria. In addition, DS conducted the acquisition of data, suggested analysis and interpretation of results and participated in designing the manuscript. VR gave support in the acquisition of data and validated the results. OS conceived the study, participated in its design and coordination. All authors reviewed and approved the final manuscript.

## Supplementary Material

Additional file 1 **Table S1.** Sexual parameters. **Table S2**: Estimated cervical cancer mortality rates by age and disease state. Table S3: Treatment patterns for each CIN/CIS stage and for genital warts. **Table S4**: Percentage of the German female population that undergo hysterectomy annually by age group. **Table S5**: Health utility values by age and gender for individuals without cervical disease or genital warts. Click here for file
