# Supplementary material for: Cost-effectiveness of vaccination with a quadrivalent HPV vaccine in Germany using a dynamic transmission model
Source: Health Econ Rev. 2012 Sep 25;2:19. doi: 10.1186/2191-1991-2-19 (PMC3575401; doi:10.1186/2191-1991-2-19)
Supplement: Additional file 1 — Table S1. Sexual parameters. Table S2: Estimated cervical cancer mortality rates by age and disease state. Table S3: Treatment patterns for each CIN/CIS stage and for genital warts. Table S4: Percentage of the German female population that undergo hysterectomy annually by age group. Table S5: Health utility values by age and gender for individuals without cervical disease or genital warts. [file 2191-1991-2-19-S1.doc]

**Additional Table 1: Sexual parameters**

| **Annual mean number of sexual partners by age group** | |
| --- | --- |
| **Age Group (years)** | **Annual mean number of sexual partners** |
| **12-14** | 0.10 |
| **15-17** | 1.00 |
| **18-19** | 2.00 |
| **20-24** | 1.90 |
| **25-29** | 1.30 |
| **30-34** | 1.30 |
| **35-39** | 1.10 |
| **40-44** | 1.10 |
| **45-49** | 1.00 |
| **50-54** | 0.90 |
| **55-59** | 0.70 |
| **60-64** | 0.40 |
| **65-69** | 0.40 |
| **70-74** | 0.30 |
| **75-79** | 0.30 |
| **80-84** | 0.30 |
| **85+** | 0.30 |
| **Sexual mixing parameters** | **Degree of assortativeness of the population by age and sexual activity group** |
| Age group mixing | 0.6 |
| Sexual activity group mixing | 0.7 |

**Additional Table 2: Estimated cervical cancer mortality rates by age and disease state**

1. Cervical cancer associated death rates Bavaria 2002-2007

| Extent of  disease | Age group | Years from date of diagnosis | Patients entered interval | Patients censored | Patients under risk | Cervical associated deaths | **Cervical associated death rate** | Survival rate (excluding non cervival associated deaths) | Standard error of survival |
| --- | --- | --- | --- | --- | --- | --- | --- | --- | --- |
| local | 0-39 | 1 | 459 | 0 | 459.0 | 1 | **0.002** | 0.998 | 0.002 |
|  | 40-49 | 1 | 560 | 5 | 557.5 | 9 | **0.016** | 0.984 | 0.005 |
|  | 50-59 | 1 | 305 | 0 | 305.0 | 4 | **0.013** | 0.987 | 0.007 |
|  | 60-69 | 1 | 203 | 2 | 202.0 | 3 | **0.015** | 0.985 | 0.009 |
|  | 70+ | 1 | 208 | 11 | 202.5 | 13 | **0.064** | 0.936 | 0.017 |
| regional | 0-39 | 1 | 143 | 3 | 141.5 | 8 | **0.057** | 0.943 | 0.019 |
|  | 40-49 | 1 | 217 | 4 | 215.0 | 21 | **0.098** | 0.902 | 0.020 |
|  | 50-59 | 1 | 174 | 2 | 173.0 | 9 | **0.052** | 0.948 | 0.017 |
|  | 60-69 | 1 | 145 | 5 | 142.5 | 16 | **0.112** | 0.888 | 0.026 |
|  | 70+ | 1 | 159 | 22 | 148.0 | 38 | **0.257** | 0.743 | 0.036 |
| distant metastases | 0-39 | 1 | 25 | 1 | 24.5 | 7 | **0.286** | 0.714 | 0.091 |
|  | 40-49 | 1 | 48 | 2 | 47.0 | 17 | **0.362** | 0.638 | 0.070 |
|  | 50-59 | 1 | 64 | 2 | 63.0 | 22 | **0.349** | 0.651 | 0.060 |
|  | 60-69 | 1 | 53 | 12 | 47.0 | 21 | **0.447** | 0.553 | 0.073 |
|  | 70+ | 1 | 73 | 10 | 68.0 | 43 | **0.632** | 0.368 | 0.058 |
| unknown | 0-39 | 1 | 198 | 2 | 197.0 | 3 | **0.015** | 0.985 | 0.009 |
|  | 40-49 | 1 | 187 | 2 | 186.0 | 8 | **0.043** | 0.957 | 0.015 |
|  | 50-59 | 1 | 114 | 5 | 111.5 | 12 | **0.108** | 0.892 | 0.029 |
|  | 60-69 | 1 | 115 | 5 | 112.5 | 16 | **0.142** | 0.858 | 0.033 |
|  | 70+ | 1 | 284 | 43 | 262.5 | 64 | **0.244** | 0.756 | 0.027 |

1. Cervical cancer associated death rates Bavaria 2002-2007 adjusted using a factor 12.5 % to meet German rate

| Extent of disease | Age group | Years from date of diagnosis | Patients entered interval | Patients censored | Patients under risk | Cervical associated deaths | **Cervical associated death rate** |  | Survival rate (ignoring non cervival associated deaths) | Standard error of survival |
| --- | --- | --- | --- | --- | --- | --- | --- | --- | --- | --- |
| local | 0-39 | 1 | 459 | 0 | 459.0 | 1 | **0.002** | **0.002451** | 0.998 | 0.002 |
|  | 40-49 | 1 | 560 | 5 | 557.5 | 9 | **0.016** | **0.018161** | 0.984 | 0.005 |
|  | 50-59 | 1 | 305 | 0 | 305.0 | 4 | **0.013** | **0.014754** | 0.987 | 0.007 |
|  | 60-69 | 1 | 203 | 2 | 202.0 | 3 | **0.015** | **0.016708** | 0.985 | 0.009 |
|  | 70+ | 1 | 208 | 11 | 202.5 | 13 | **0.064** | **0.072222** | 0.936 | 0.017 |
| regional | 0-39 | 1 | 143 | 3 | 141.5 | 8 | **0.057** | **0.063604** | 0.943 | 0.019 |
|  | 40-49 | 1 | 217 | 4 | 215.0 | 21 | **0.098** | **0.109884** | 0.902 | 0.020 |
|  | 50-59 | 1 | 174 | 2 | 173.0 | 9 | **0.052** | **0.058526** | 0.948 | 0.017 |
|  | 60-69 | 1 | 145 | 5 | 142.5 | 16 | **0.112** | **0.126316** | 0.888 | 0.026 |
|  | 70+ | 1 | 159 | 22 | 148.0 | 38 | **0.257** | **0.288851** | 0.743 | 0.036 |
| distant metastases | 0-39 | 1 | 25 | 1 | 24.5 | 7 | **0.286** | **0.321429** | 0.714 | 0.091 |
|  | 40-49 | 1 | 48 | 2 | 47.0 | 17 | **0.362** | **0.406915** | 0.638 | 0.070 |
|  | 50-59 | 1 | 64 | 2 | 63.0 | 22 | **0.349** | **0.392857** | 0.651 | 0.060 |
|  | 60-69 | 1 | 53 | 12 | 47.0 | 21 | **0.447** | **0.502660** | 0.553 | 0.073 |
|  | 70+ | 1 | 73 | 10 | 68.0 | 43 | **0.632** | **0.711397** | 0.368 | 0.058 |
| unknown | 0-39 | 1 | 198 | 2 | 197.0 | 3 | **0.015** | **0.017132** | 0.985 | 0.009 |
|  | 40-49 | 1 | 187 | 2 | 186.0 | 8 | **0.043** | **0.048387** | 0.957 | 0.015 |
|  | 50-59 | 1 | 114 | 5 | 111.5 | 12 | **0.108** | **0.121076** | 0.892 | 0.029 |
|  | 60-69 | 1 | 115 | 5 | 112.5 | 16 | **0.142** | **0.160000** | 0.858 | 0.033 |
|  | 70+ | 1 | 284 | 43 | 262.5 | 64 | **0.244** | **0.274286** | 0.756 | 0.027 |

The Cancer Registry of Bavaria provided us with the cervical associated death rates in Bavaria, since there is no central registry in Germany that accounts for the whole country. Bavaria shows a lower CC death rate than expected for the whole country: Bavaria has an EU standardized rate of 2.2, the world standardized rate is 1.6. In contrast to this, the expected EU standardized rate for Germany is 2.5, the world standardized rate for Germany is 1.8 (). Thus, we used the ratio of 12.5% to adjust the cancer death relation Bavaria to Germany.

Additional table 3: Treatment patterns for each CIN/CIS stage and for genital warts

| **CIN/CIS stage** | **Percentage treated** | **Sources** |
| --- | --- | --- |
| **CIN1** | 12 | Petry et al 2008 |
| **CIN2** | 40 |
| **CIN3** | 100 |
| **CIS** | 100 | Assumption |
| **Genital warts** | 74 | Hillemanns et al 2008 |

Additional table 4: Percentage of the German female population that undergo hysterectomy annually by age group .

| **Age Group (years)** | **Percentage undergoing hysterectomy annually** |
| --- | --- |
| **12-14** | 0.0000923 |
| **15-17** | 0.000364 |
| **18-19** | 0.0002771 |
| **20-24** | 0.002829 |
| **25-29** | 0.01909687 |
| **30-34** | 0.10133664 |
| **35-39** | 0.348383814 |
| **40-44** | 0.7619662 |
| **45-49** | 0.92468191 |
| **50-54** | 0.58811371 |
| **55-59** | 0.33585563 |
| **60-64** | 0.31436301 |
| **65-69** | 0.35043554 |
| **70-74** | 0.34408074 |
| **75-79** | 0.31113046 |
| **80-84** | 0.21041396 |
| **85+** | 0.11104291 |

Additional table 5: Health utility values by age and gender for individuals without cervical disease or genital warts

| **Age Group (years)** | **Male** | **Female** |
| --- | --- | --- |
| **12-14** | 0.96 | 0.9330 |
| **15-17** | 0.96 | 0.9330 |
| **18-19** | 0.96 | 0.9330 |
| **20-24** | 0.96 | 0.9330 |
| **25-29** | 0.96 | 0.9330 |
| **30-34** | 0.96 | 0.9330 |
| **35-39** | 0.96 | 0.9330 |
| **40-44** | 0.928 | 0.882 |
| **45-49** | 0.928 | 0.882 |
| **50-54** | 0.928 | 0.882 |
| **55-59** | 0.928 | 0.882 |
| **60-64** | 0.878 | 0.8 |
| **65-69** | 0.878 | 0.8 |
| **70-74** | 0.878 | 0.8 |
| **75-79** | 0.878 | 0.8 |
| **80-84** | 0.878 | 0.8 |
| **85+** | 0.878 | 0.8 |
